# A ferret brain slice model of oxygen–glucose deprivation captures regional responses to perinatal injury and treatment associated with specific microglial phenotypes

**DOI:** 10.1002/btm2.10265

**Published:** 2021-11-23

**Authors:** Thomas R. Wood, Kate Hildahl, Hawley Helmbrecht, Kylie A. Corry, Daniel H. Moralejo, Sarah E. Kolnik, Katherine E. Prater, Sandra E. Juul, Elizabeth Nance

**Affiliations:** ^1^ Department of Pediatrics, Division of Neonatology University of Washington Seattle Washington USA; ^2^ Center on Human Development and Disability University of Washington Seattle Washington USA; ^3^ Department of Chemical Engineering University of Washington Seattle Washington USA; ^4^ Department of Neurology University of Washington Seattle Washington USA; ^5^ Department of Bioengineering University of Washington Seattle Washington USA

**Keywords:** ferret, machine learning, microglia, neonatal, neuroprotection, organotypic brain slice, therapeutic screening

## Abstract

Organotypic brain slice models are an ideal technological platform to investigate therapeutic options for hypoxic‐ischemic (HI) brain injury, a leading cause of morbidity and mortality in neonates. The brain exhibits regional differences in the response to HI injury in vivo. This can be modeled using organotypic brain slices, which maintain three‐dimensional regional structures and reflect the regional differences in injury response. Here, we developed an organotypic whole hemisphere (OWH) slice culture model of HI injury using the gyrencephalic ferret brain at a developmental stage equivalent to a full‐term human infant in order to better probe region‐specific cellular responses to injury. Each slice encompassed the cortex, corpus callosum, subcortical white matter, hippocampus, basal ganglia, and thalamus. Regional responses to treatment with either erythropoietin (Epo) or the ketone body acetoacetate (AcAc) were highly heterogenous. While both treatments suppressed global injury responses and oxidative stress, significant neuroprotection was only seen in a subset of regions, with others displaying no response or potential exacerbation of injury. Similar regional heterogeneity was seen in the morphology and response of microglia to injury and treatment, which mirrored those seen after injury in vivo. Within each region, machine‐learning‐based classification of microglia morphological shifts in response to injury predicted the neuroprotective response to each therapy, with different morphologies associated with different treatment responses. This suggests that the ferret OWH slice culture model provides a platform for examining regional responses to injury in the gyrencephalic brain, as well as for screening combinations of therapeutics to provide global neuroprotection after injury.

## 1 INTRODUCTION

Perinatal asphyxia and subsequent hypoxia‐ischemia (HI), characterized by a lack of blood flow and oxygenation to the brain, is a leading cause of morbidity and mortality in neonates. Affecting 2–4 per 1000 term births, HI can lead to severe neurodevelopmental impairment and disability, including cerebral palsy and epilepsy.^1^ Although therapeutic hypothermia (TH) has reduced the incidence of death and disability in term infants who experience significant HI and subsequent hypoxic‐ischemic encephalopathy (HIE), at least 30% of these infants will still experience a poor outcome.^2^ Therefore, there is a need for models of perinatal brain injury that allow for study of the pathophysiological changes underlying HI as well as for the development of new therapeutic platforms. The ferret is increasingly being used to study perinatal brain injury due to its structural and developmental similarities with the human brain.^3^, ^4^, ^5^ Ferrets have a more comparable white to gray matter ratio as well as a gyrencephalic cortex, making them more suitable than traditional rodent models to simulate human brain injury. In addition, the ferret undergoes a number of key neurodevelopmental processes postnatally, including cortical folding and white matter maturation. Access to these neurodevelopmental processes postnatally allows for easier intervention during these events, making ferrets ideal for the study of neonatal brain injury.

Brain injury from HI is complex and heterogeneous across brain structures. Following immediate energy failure from HI, secondary insult occurs from an accumulation of excitotoxic, inflammatory, and oxidative processes post‐reperfusion.^6^, ^7^ Ex vivo organotypic brain slice models are an ideal technological platform to study these injury mechanisms; slice models maintain three‐dimensional cellular architecture and cell function, and when performed as organotypic whole hemisphere (OWH) slice models,^8^, ^9^ retain distinct cerebral structures, which are known to have differing responses to injury in vivo. Slice cultures, particularly OWH cultures, therefore, confer certain advantages over traditional primary cell cultures that lack complex structure and multicell type interactions. In addition, ex vivo preclinical models allow for higher throughput screening of therapeutics prior to potentially costly in vivo efficacy studies while also enabling collection of high‐fidelity and high‐dimensionality data amenable to more advanced data science techniques including machine‐learning‐based analytical and predictive tools. While rodent cortical and hippocampal slices, and more recently whole hemisphere models, have been used in neurodevelopmental science, slice models have not been established using the ferret.

In this study, we develop a term‐equivalent ferret OWH brain slice model of oxygen‐glucose deprivation (OGD) to better probe injury responses seen in our recently described neonatal in vivo brain injury model in the developing ferret.^4^ We then use the OWH slice culture model to investigate the potential for studying both global and regional mechanisms and cellular responses to injury and therapy under conditions that mimic the pathophysiological causes of HIE.

## 2 METHODS

### 2.1 Animal care and ethics

This study was performed in strict accordance with the recommendations in the Guide for the Care and Use of Laboratory Animals of the National Institutes of Health. All of the animals were handled according to approved institutional animal care and use committee (IACUC) protocols #3328‐06 and #3328–07 of the University of Washington. The University of Washington has an approved Animal Welfare Assurance (#A3464‐01) on file with the National Institute of Health Office of Laboratory Animal Welfare (OLAW), is registered with the United States Department of Agriculture (USDA, certificate #91‐R‐0001), and is accredited by American Association for Accreditation of Laboratory Animal Care International. Every effort was made to minimize suffering. Ferret jills with previously cross‐fostered kits were purchased from Marshall BioResources (North Rose, NY, USA) and arrived at the facility at or before postnatal day (P) 15. Animals were maintained in a centralized vivarium and had ad libitum access to food and water. Standard housing conditions included a 16 h light–8 h dark cycle with a room temperature range of 61–72 °F (16–22°C), humidity of 30%–70%, and 10–15 fresh air changes per hour.

### 2.2 Regional Iba‐1 responses in an in vivo ferret model of inflammation‐sensitized perinatal brain injury

On the morning of P17, ferret kits were removed from the nest, weighed, and randomized to either the nonsurgical control or the hypoxic‐ischemic‐hyperoxic (HIH)‐exposed group, as previously described.^4^ Briefly, controls and HIH‐exposed ferrets received 3 μl/g saline vehicle or 3 mg/kg lipopolysaccharide (LPS, ultrapure from *E. coli* 055:B5, List Biological, CA) intraperitoneally, respectively. Animals that received LPS underwent a bilateral carotid ligation under isoflurane anesthesia (3%–5%) plus buprenorphine (0.05 mg/kg) analgesia. The left carotid artery (LCA) was ligated twice with silk suture (5–0, Fine Science Tools, Foster City, CA) and then transected between the two ligations. The right carotid artery (RCA) was temporarily occluded with umbilical tape (1/8in, GF Health Products, Atlanta, GA). The incision site was closed with wound clips, and the animals were taken to a separate room to recover. After a 30 min rest period, kits were placed in a prewarmed chamber and exposed to hypoxia (9% oxygen for 30 min) followed by hyperoxia (80% oxygen for 30 min), then hypoxia again (9% oxygen for 30 min). Internal body temperature of two sentinel ferrets was closely monitored to maintain normothermia (target rectal temperature 37°C). After the second period of hypoxia, the umbilical tape on the RCA was removed in a second surgical procedure. After 1 h rest with the jills, HIH‐exposed animals received either subcutaneous saline vehicle (Veh) or Epo (2000 IU/kg) s.c. at 0 h, 24 h, 48 h, and 7 days. For 6 h after the first dose, animals were placed in a water bath to ensure normothermia (36–37°C). At P42, kits were euthanized and perfusion fixed with phosphate‐buffered saline (PBS) followed by 10% neutral buffered formalin. Coronal slices at the level of the caudate nucleus were taken from each brain, embedded in paraffin, and 4 μm sections were prepared for immunohistochemistry (IHC). Iba‐1 (1:1500 dilution, WAKO Chemicals USA, 019‐19741) IHC was performed at the University of Washington Harborview Medical Center (HMC) Histology IHC lab, as previously described.^5^ Image analysis was performed using whole slide digital images and automated image analysis. All slides were scanned in bright field with a 20× objective using a Nanozoomer Digital Pathology slide scanner (Hamamatsu; Bridgewater, New Jersey) and the digital images were imported into Visiopharm software (Hoersholm, Denmark) for quantitative analysis. Regions of interest (ROIs) were manually traced on both the left and right hemisphere and were taken from brain regions that included the thalamus, subcortical white matter, dorsal cortex, hippocampus, as well as the corpus callosum. Therefore, “ROI” refers to the specific field of view from which data were collected, and “region” refers to the specific area or structure of the brain that the ROI was taken from. Visiopharm was then trained to label positive staining and the background tissue counter stain using a project and stain‐specific configuration based on threshold pixel values. Images were processed in batch mode using this configuration to generate positively stained to unstained tissue ratios, presented in arbitrary units (au).

### 2.3 OWH brain slice preparation

At postnatal days 21–23, equivalent to term human gestation, ferret kits were deeply anesthetized using 5% isoflurane and then administered an overdose of pentobarbital (120–150 mg/kg i.p.). Animals were then quickly decapitated using a guillotine, and the whole brain was removed and placed into ice‐cold dissecting media (0.64% w/v glucose, 100% HBSS [Hank's Balanced Salt Solution], 1% penicillin–streptomycin). A total of 300 μm whole‐hemisphere live slices were obtained using a Leica Vibratome. Slices were transferred to 35‐mm, 0.4‐μm‐pore membrane inserts in a six‐well plate and cultured in 1 ml of either 5% or 10% heat‐inactivated horse‐serum slice culture media (SCM) (50% MEM [Minimum Essential Media], 45/40% HBSS, 1% GlutaMAX, and 1% penicillin–streptomycin). During initial development, slice health was compared under normal control (NC) conditions using both 5% and 10% serum. Following two day in vitro (DIV), slice health was assessed by lactate dehydrogenase (LDH) release and nuclear propidium iodide (PI) accumulation. Minimal PI uptake and LDH release were observed in the 5% NC slices. Increasing serum from 5% to 10% did not change glutathione levels but resulted in artificially reduced LDH release due to high background signal (Supplemental Figure 1). Therefore, OGD experiments used 5% serum in the NC group. For RNA analysis, two slices were placed adjacently on a single membrane. Slices rested in SCM overnight for approximately 24 h in a sterile CO_2_ incubator at constant temperature (37°C), humidity, and CO_2_ (5%). All slices not subjected to OGD were considered to be NC slices and were maintained in SCM with 5% heat‐inactivated horse serum for the duration of the study.

### 2.4 Oxygen–glucose deprivation

After one DIV, slices were subjected to a period of OGD; SCM was replaced with glucose‐free OGD media (150 mM NaCl, 2.8 mM KCl, 1 mM CaCl_2_, and 10 mM HEPES (4‐(2‐hydroxyethyl)‐1‐piperazineethanesulfonic acid) in DI H_2_0).^10^ OGD media was prewarmed to 37°C and bubbled for 5 min with N_2_ at a flow rate of 3 L/min. The slices were placed into a sealable chamber. The chamber was flushed with N_2_ for 10 min at a flow rate of 5 L/min at room temperature and placed into an incubator at 37°C for either 1 h or 2 h. Slices were then removed from the chamber and the media was replaced with 5% SCM or, if applicable, SCM containing 1 IU/ml erythropoietin (Epo) or 3 mM acetoacetate (AcAc), which are concentrations that have previously been shown to be neuroprotective in vitro.^11^, ^12^ For all studies, the end of OGD incubation was defined as time t = 0 h. The slices were cultured for an additional 24 h after OGD. SCM was removed and added in the 5% NC and 10% NC slices at the same intervals as 2 h OGD slices to match the number of media changes. Supernatant was collected during all media changes.

### 2.5 LDH assay

Supernatant collected at the end of OGD (time t = 0 h) and at the end of culturing (t = 24 h) was immediately stored at −80°C. This supernatant was thawed at room temperature prior to running the LDH assay (Cayman Chemical). LDH is an enzyme released from cells upon membrane degradation in response to cytotoxicity. Through a series of coupled enzymatic reactions, LDH can be converted to formazan, which absorbs in the 490–520 nm range. A total of 100 μl of thawed supernatant was seeded in triplicate into a 96 well plate. A total of 100 μl of chilled LDH reaction buffer was added to each well. The plate was incubated at 37°C for 30 min. Absorbance was measured at 490 nm (A_490_) on a UV–Vis Spectrophotometer. A_490_ was summed for the t = 0 h and t = 24 h time points to give a measure of cumulative LDH release. Supernatant was tested for *n =* 12 slices per group with an equal sex split. Values are reported as the A_490_ value subtracted from the background absorbance of the media.

### 2.6 PI staining

Slices were stained at t = 24 h with 5 μg/ml PI in 5% SCM at 37°C ‐ 900 μl was placed below the insert and 100 μl was added directly on top of the slice. PI is a fluorescent stain (bound excitation maximum of 617 nm) that intercalates with nucleic base pairs but cannot permeabilize into healthy, live cells. Supernatant was removed after 45 min and slices were washed with 5% SCM. After 2 h, the supernatant was again removed and replaced for an overnight wash. Slices were then washed with 1× phosphate buffered saline (PBS), fixed in 10% buffered formalin, and stored in 1× PBS at 4°C. Prior to imaging, the slices were stained with 1 ml of 5 μg/ml DAPI (4′,6‐diamidino‐2‐phenylindole, Invitrogen) in 1× PBS for 5 min then washed twice in 1× PBS. Representative images were obtained in six different regions using a Nikon confocal microscope with a 40× objective. For each condition, *n =* 8–10 slices were imaged with an equal sex split. Six ROIs of identical size were counted in each slice, encompassing the cortex, thalamus, corpus callosum, subcortical white matter, hippocampus, and basal ganglia. Cells positive for PI in the DAPI‐stained nuclear region were identified and counted manually in the entire field of view for each ROI. Cell counting was performed by a team of researchers blinded to treatment group assignments.

### 2.7 Mitochondrial morphology staining

Slices were stained at time t = 24 h with 500 nM MitoTracker Deep Far Red FM (ThermoFisher) in 5% SCM at 37°C (900 μl was placed below the insert, and 100 μl was added directly on top of the slice). Supernatant was removed after 45 min and slices were washed in 5% SCM, fixed in 10% buffered formalin, and stored in 1x PBS in the same manner as PI‐stained slices. Prior to imaging, the slices were stained with 1 ml of 5 μg/ml DAPI in 1× PBS for 5 min then washed twice in 1× PBS. Representative images were obtained using a Nikon confocal microscope with a 60× objective. The images were selected from *n* = 2 (AcAc only) or *n* = 4 slices (all other conditions) with an equal sex split for each condition.

### 2.8 Iba‐1 and Caspase‐3/7 staining

Slices were stained at time t = 0 h with 5 μM CellEvent™ Caspase‐3/7 Green Detection Reagent. Supernatant was removed after 45 min and slices were washed in 5% SCM, fixed in 10% buffered formalin, and stored in 1× PBS in the same manner as PI‐stained slices. Prior to imaging, the slices were co‐stained using a primary antibody for microglia (Wako rabbit anti‐Iba1) at 1:250 in PBS++ (0.01% Triton‐X, normal goat serum) for 6 h at room temperature. After 6 h, slices were washed twice with PBS and the secondary antibody solution (AF‐488 IgG goat anti‐rabbit, Invitrogen) was added to the slices at 1:500 in PBS for an additional hour at room temperature. Slices were washed twice in PBS and stained with 5 μg/ml DAPI in PBS for 5 min before final two washes in PBS. Representative images from *n* = 6–8 slices (equal sex split) were obtained using a Nikon confocal microscope. Z‐stack images were obtained using a 40× objective.

### 2.9 Microglial morphology analysis

Z‐stack images from each ROI of Iba‐1‐stained slices were converted from.nd2 file format to.tiff file format. Images were then split by color channel, green for Iba‐1 and blue for DAPI. Images from both color channels were split into four equal quadrants in order to increase image number for training and testing split.^13^ Converted images underwent a train:test split at a ratio of 80:20 ensuring at least two images from each sex, region, and treatment combination remained after the split. Cells in images were segmented using the Otsu threshold from Sci‐kit Image in Python.^14^ Objects smaller than 25 pixels were removed and holes were filled. Segmented cell images were saved as .png files. The training images were then fed to the Visual Aided Morpho‐Phenotyping Image Recognition (VAMPIRE) methodology to train a model with a shape mode (SM) number of five and registration coordinates variable set to 50.^15^ Five SMs where chosen to capture biological variation while remaining computationally efficient. The test images were then run on this model to classify all cells into the five SMs determined during training. Three main morphology parameters of each cell were also determined—perimeter, circularity, and area coverage, defined as the number of pixels within the segmented cell outline. Total number of Iba‐1‐stained cells per brain structure ROI per slice was estimated by multiplying the total cell counts in the 20% of test images by 5.

### 2.10 Glutathione assay

At t = 24 h, slices were carefully removed from the membrane and transferred to 1 ml centrifugation tubes and rinsed quickly with normal saline. After the saline was removed using a pipette, slices were flash‐frozen using dry ice and 2‐methylbutane. Samples were stored at −80°C prior to processing. The ratio of reduced glutathione (GSH) to oxidized glutathione (GSSG) was measured using the GSH/GSSG Ratio Detection Assay Kit (Fluorometric‐Green; Abcam, ab138881) according to the manufacturer's protocol. Serial dilutions of both the GSH and GSSG stock standards were prepared and run alongside 1:10 dilutions of brain lysates after incubating at room temperature for 30 min. The fluorescence intensity was assessed using a BioTek fluorescence plate reader at 490 nm emission/520 nm excitation. *n* = 8–10 slices were analyzed, with at least *n* = 4 slices of each sex.

### 2.11 RNA

At t = 24 h, slices were gently removed from the membrane and placed into RNALater (Invitrogen) and stored at −20°C. RNA extractions were performed based on the manufacturer protocol for TRIzol (Invitrogen). Slices were rinsed in H_2_O and 1 ml of TRIzol was added. The slices were homogenized in the TRIzol and then chloroform was added, followed by a brief centrifugation to induce phase separation. The aqueous layer was transferred to a new tube and isopropanol was added to precipitate RNA, followed by a series of ethanol washes. RNA concentration was determined using a Nanodrop. The RNA was converted to cDNA using a High Capacity RNA to cDNA kit (Applied Biosystems) and diluted to 20 ng/μl. Real‐time PCR (RT‐PCR) was performed using the PowerUp SYBR Green Master Mix (AppliedBiosystems, A25741) according to the manufacturer's protocol. Primers developed in‐house to target specific ferret genes are shown in Table 1. The following mRNA profiles were measured: tumor necrosis factor‐alpha (TNFα), interleukin‐6 (IL‐6), IL‐1 beta (IL‐1β), IL‐8, IL‐10, prostaglandin E synthase 2 (PTGES2), glutamate‐cysteine ligase modifier subunit (GCLM), heme oxygenase 1 (HMOX1), ChaC glutathione specific gamma‐glutamylcyclotransferase 1 (CHAC1), and solute carrier family 7 member 11 (SLC7A11). ∆Ct values were calculated in comparison to housekeeping gene glyceraldehyde‐3‐phosphate dehydrogenase (GAPDH). ∆∆Ct values were calculated using NC slices as reference values. Profiles are reported for *n* = 6 slices (equal gender split) for all conditions.

**TABLE 1:** Ferret primers

Target	Forward primer sequence	Reverse primer sequence
GAPDH	TGCGGCCAAGGCAGTAG	AGGCCATGCCAGTGAGCTT
TNFα	AGCCAGGAGAGACAGAAG	CAGCTGGGTGATGGTAAAG
IL‐6	GGCTCAACTGAGGGTGATAAA	GGGCCAGGAGACGAATG
IL‐1β	GGACTGCAAATTCCAGGACATAA	TTGGTTCACACTAGTTCCGTTGA
IL‐8	GTTCAGAACTCCGATGCC	GGCCACTGTCAATCACTC
IL‐10	GTATGATGCCTTCCTGTCCTTC	CATGCCTAGTGGAGGCAATC
PTGES2	AATGACCCGGCCTCTGA	GGAAACAGCCAGAGTCTCATC
GCLM	GGGAACCTGCTCAACTGG	GTCTCTCACCTCCTCGCT
HMOX1	CTCCAGCATTCGGAGGAAC	GGGAGGGTGAACAAGGG
CHAC1	GGTTCTGGTGACAGCTGATT	CAGGCTGAGGAGGAAAGAAA
SLC7A11	CCATCTTCTGTCTCTGGAACTG	GGAAAGGGCAACCATGAAGA

### 2.12 Statistical analysis

Statistical analysis was performed in GraphPad Prism version 9.1.0 (GraphPad Software, San Diego, CA, USA) and R version 3.6.3 (Vienna, Austria).^16^ Graphs for PI, LDH, GSH, PCR, and quantitative IHC results are displayed as median with interquartile range. In column graphs, all data points are shown, with males and females represented by circles and triangles, respectively. For PCR analysis, *n* = 6 slices were used per condition and all samples were run in triplicate and the mean taken to provide the final value. For LDH assays, supernatant from *n* = 12 slices was tested in triplicate and the mean was taken to provide the final value. PI images were acquired for *n* = 8–10 slices per condition in each brain region. All data were assumed to not be normally distributed. As such, pairwise group comparisons were determined a priori, with comparisons between OGD and NC, and OGD and the two treatment groups performed using a Kruskall–Wallace test followed by Dunn's post hoc correction for multiple comparisons. Microglial cell morphology characteristics were compared across all regions and groups, as well as between VAMPIRE‐derived SMs, using the same method. Odds for the likelihood of a microglia displaying a certain SM were determined using contrasts based on sex and region * group interactions using the estimated marginal means R package. The degree of neuroprotection provided by Epo or AcAc by region was calculated based on relative change in median number of cells positive for PI compared to the OGD 2 h group. This degree of neuroprotection was correlated with similar relative changes in microglial morphology in Iba‐1‐stained slices using generalized linear models to determine whether region‐specific responses to therapy were associated with certain microglial morphological changes after injury. Regional changes in proportion of the five SMs were used to predict relative changes in injury and neuroprotection by both Epo and AcAc using logistic regression to determine odds ratios (ORs) with 95% confidence interval (CI) and area under the receiver operating curve (AUROC). All *p*‐values of <0.05 were considered statistically significant.

## 3 RESULTS

### 3.1 Ex vivo model development and cytotoxicity based on in vivo regional responses to injury and treatment

**FIGURE 1:**
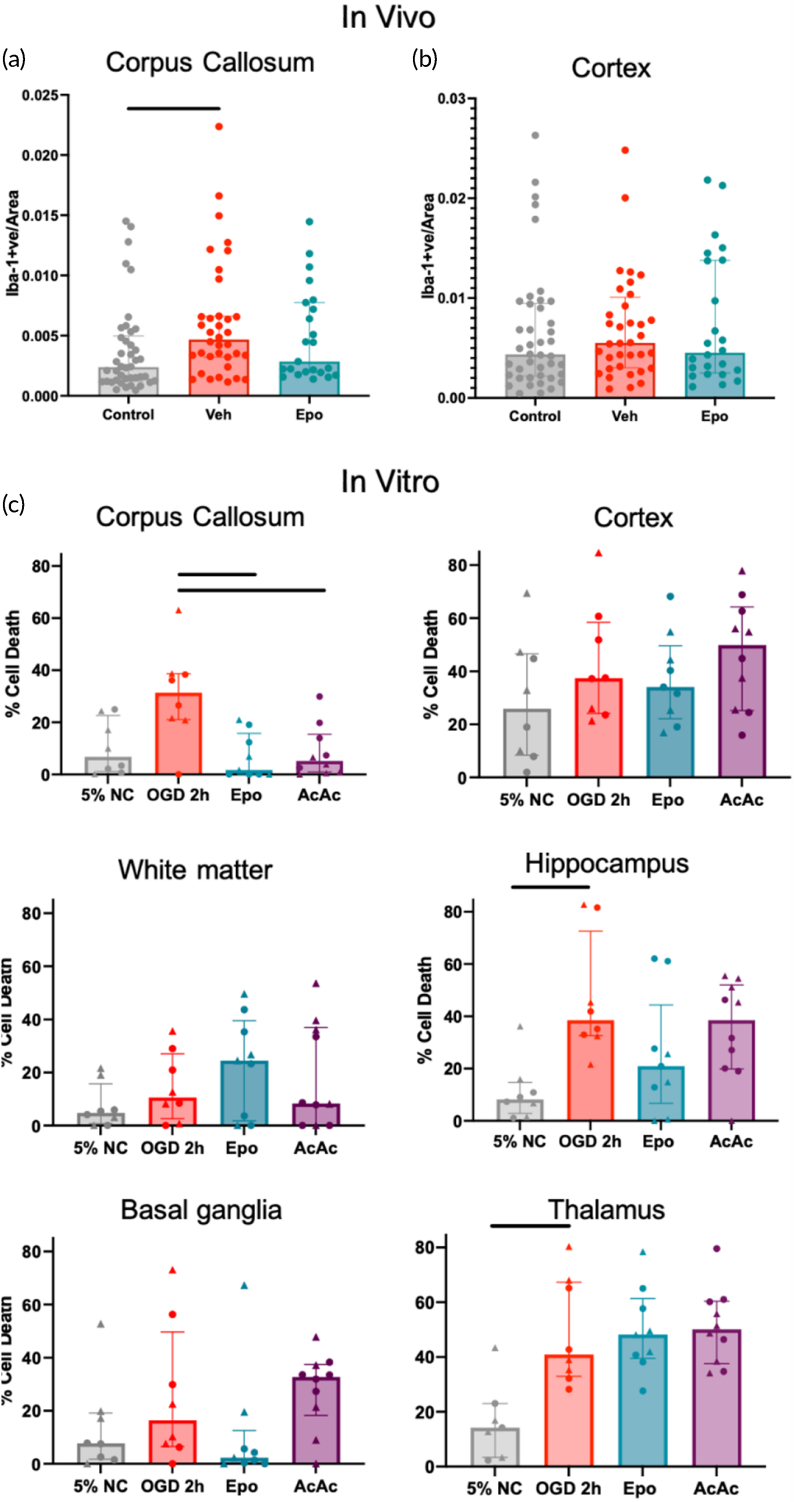
Regional myeloid cell staining intensity and cell death in vitro mimics changes seen in in vivo injury. Quantitative immunohistochemistry of Iba‐1 staining in the corpus callosum (a) and cortex (b) of ferrets at P42 after inflammation‐sensitized HI injury at P17. Iba‐1 staining intensity in the cortex of control animals at P42 was around double that seen in the corpus callosum (median 0.0044 au vs. 0.0024 au). In the corpus callosum, injury in vehicle‐treated animals significantly increased Iba‐1 staining intensity (0.0047 au vs. 0.0024 au, *p* = 0.04), which was normalized by treatment with Epo. By comparison, injury resulted in a much smaller nonsignificant relative increase in Iba‐1 staining in the cortex (0.0055 au vs. 0.0044 au), with no significant effect of Epo. (c) Regional absolute percent of PI‐positive cells per slice/condition. OGD significantly increased injury in the hippocampus and thalamus, with significant neuroprotection by treatment seen in the corpus callosum. Females are represented by ▲ markers and males are represented by ● markers. Line indicates significant difference (*p* < 0.05) with Kruskal–Wallace test after adjustment for multiple comparisons

In the in vivo injury model, Iba‐1 staining intensity in the cortex of control animals at P42 was around double that seen in the corpus callosum (median 0.0044 au vs. 0.0024 au). In the corpus callosum, injury in vehicle‐treated animals significantly increased Iba‐1 staining intensity (0.0047 au vs. 0.0024 au, *p* = 0.04), with the staining intensity then not different from control after treatment with Epo (Figure 1a). By comparison, injury resulted in a much smaller nonsignificant relative increase in Iba‐1 staining in the cortex (0.0055 au vs. 0.0044 au), with no significant effect of Epo (Figure 1b).

**FIGURE 2:**
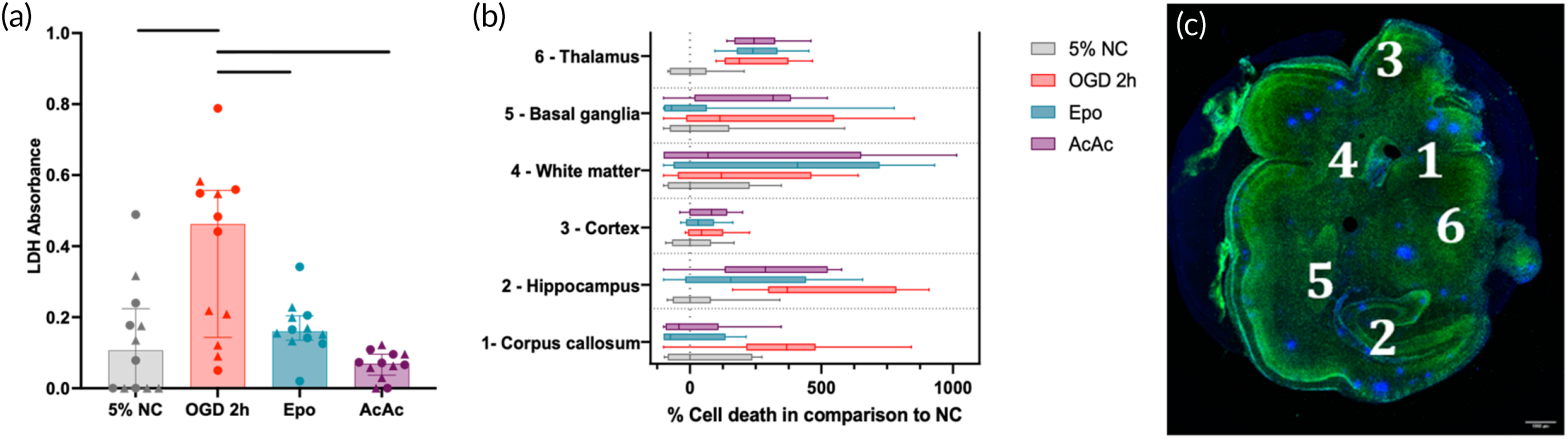
(a) Total lactate dehydrogenase (LDH) in culture media by treatment group in slices. Oxygen–glucose deprivation (OGD) 2 h significantly increased LDH release, which was normalized by treatment with both Epo and AcAc. (b) Box plots of regional percent of PI‐positive cells compared to control, normalized to the median LDH level in control slices, with a representative image of numbered regions shown in (c) Green is NeuN, with cell nuclei in blue (DAPI). Larger spots of blue are staining artifact. Line indicates significant difference (*p* < 0.05) with Kruskal–Wallace test after adjustment for multiple comparisons

To simulate injury treatment and response seen in our in vivo model of hypoxic–ischemic injury, slices were subjected to either a 1 h or 2 h period of OGD after 1 *DIV*. LDH release and cellular glutathione levels increased and decreased, respectively, with increasing OGD time, and significant differences compared to NC were only seen in the 2 h OGD group (Supplemental Figure 1). Slices that underwent 2 h of OGD had a 4.3‐fold increase in LDH release compared to NC (Figure 2a), with an associated increase in nuclear PI (Supplemental Figure 2). Compared to a cell lysis control (Triton X), 2 h of OGD resulted in about 20% total LDH release (Supplemental Figure 1). Therefore, all slices receiving therapies were exposed to 2 h of OGD prior to treatment.

### 3.2 Global and regional responses to Epo and AcAc administration

Slices were treated with either Epo or AcAc immediately following the 2 h OGD period to determine whether the slice model could be used to evaluate the effect of potential therapies on cellular health. Treatment with both therapies returned the amount of LDH released to similar level of that seen from NC slices (Figure 2a), though treatment with Epo resulted in a greater reduction in PI‐positive nuclei compared to AcAc (Supplemental Figure 2). Regional cytotoxicity, as determined by PI positive cell counts normalized to the NC group, is shown in Figure 2c. OGD increased PI staining in all regions, with notable region‐specific responses to Epo and AcAc. Both Epo (*p* = 0.005) and AcAc (*p* = 0.012) significantly reduced PI staining in the corpus callosum, whereas Epo alone reduced PI staining in the hippocampus (*p* = 0.04) and basal ganglia (*p* = 0.07). Neither therapy was protective in the cortex or thalamus, with nonsignificant increases in PI staining seen as a result of Epo or AcAc in the subcortical white matter and basal ganglia, respectively. Although PI results are displayed including sex for each datapoint for illustrative purposes, statistical power was not sufficient to analyze data based on region * treatment * sex analyses for PI staining. Normalized regional responses to injury and treatment, and a representative slice image depicting regions are shown in Figure 2b,c.

### 3.3 Cellular glutathione levels

**FIGURE 3:**
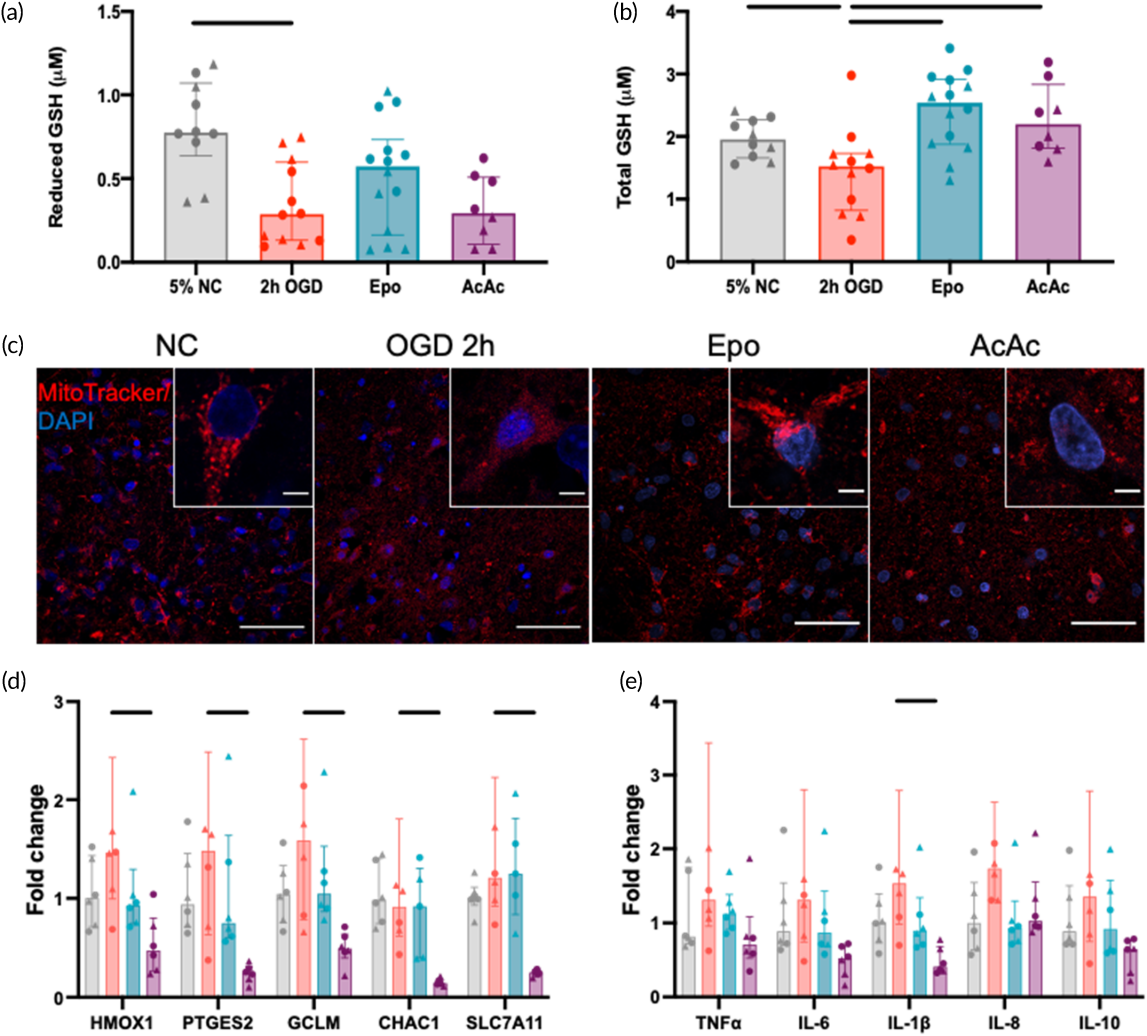
Glutathione (GSH), mitochondrial responses, and RNA expression responses to oxygen–glucose deprivation (OGD) and therapy. (a) Reduced GSH in slices by treatment group. OGD 2 h resulted in significantly lower levels of reduced GSH, which was normalized by Epo but not AcAc. (b) Total GSH in slices by treatment group. OGD 2 h resulted in significantly lower total GSH, which was significantly increased by both Epo and AcAc. (c) Representative images of mitochondria by treatment group. Widespread fission was seen in the OGD 2 h group. Mitochondrial structure was largely normalized by Epo, with an intermediate phenotype in the AcAc group. Scale bars are 50 μm and 5 μm (inset images). **(**d,e) qPCR of transcripts associated with responses to oxidative stress (left) and inflammation (right). OGD 2 h (red) tended to increase the expression of HMOX1, PTGES2, GCLM, TNF‐α, IL‐6, IL‐1β, and IL‐8. Although Epo also tended to normalize these responses, only AcAc resulted in significant decreases in expression, generally to below the levels seen in control slices. One slice in the OGD group exhibited fold‐changes in multiple transcripts that were above the limits of the visible axes but was still included in statistical analyses. Line indicates significant difference (*p* < 0.05) by Kruskal–Wallace test adjusted for multiple comparisons

The impact of OGD on cellular redox status was qualitatively assessed through an analysis of glutathione (GSH) levels. The concentration of reduced glutathione decreased by 63% with OGD exposure, which was nonsignificantly reversed with Epo treatment but not by AcAc (Figure 3a). Exposure to OGD also reduced total GSH by 22% compared to NC, with significant increases seen in both the Epo and AcAc groups compared to OGD (Figure 3b).

### 3.4 Mitochondrial morphology

Using MitoTracker Deep Far Red to qualitatively assess mitochondrial structure in the corpus callosum, NC cells had a highly structured morphology, with mitochondria clearly organized around the nucleus (Figure 3c). Following 2 h of OGD, mitochondria appeared smaller and fragmented, with a widely dispersed MitoTracker signal. Epo‐treated slices showed a return to NC mitochondrial morphology, while AcAc treatment resulted in only a partial normalization of mitochondrial morphology.

### 3.5 Expression of markers associated with responses to oxidative stress and inflammation

For quantitative polymerase chain reaction analyses, slices were collected 24 h after OGD exposure. There was a nonsignificant approximately 50% increase in antioxidant markers HMOX1, PTGES2, and GCLM following OGD exposure (Figure 3D). Median fold expression of all oxidative stress markers was similar to NC levels, but none were significantly lower than the OGD group. By comparison, treatment with AcAc caused a significant reduction for all oxidative stress markers (HMOX1, PTGES2, GCLM, CHAC1, and SLC7A11), relative to OGD (Figure 3d). Similar to the genes associated with oxidative stress, cytokine expression (TNF‐α, IL‐6, IL‐1β, IL‐8, and IL‐10) increased by 50%–75% in the OGD group compared to NC, but due to significant variability, these differences were not significant (Figure 3e). Epo treatment also appeared to normalize cytokine expression, but none of the individual comparisons were statistically significant. In general, AcAc resulted in greater reductions in cytokine expression, but only the effect on IL‐1β was statistically significant (Figure 3e).

### 3.6 Microglial number responses to injury and treatment

**FIGURE 4:**
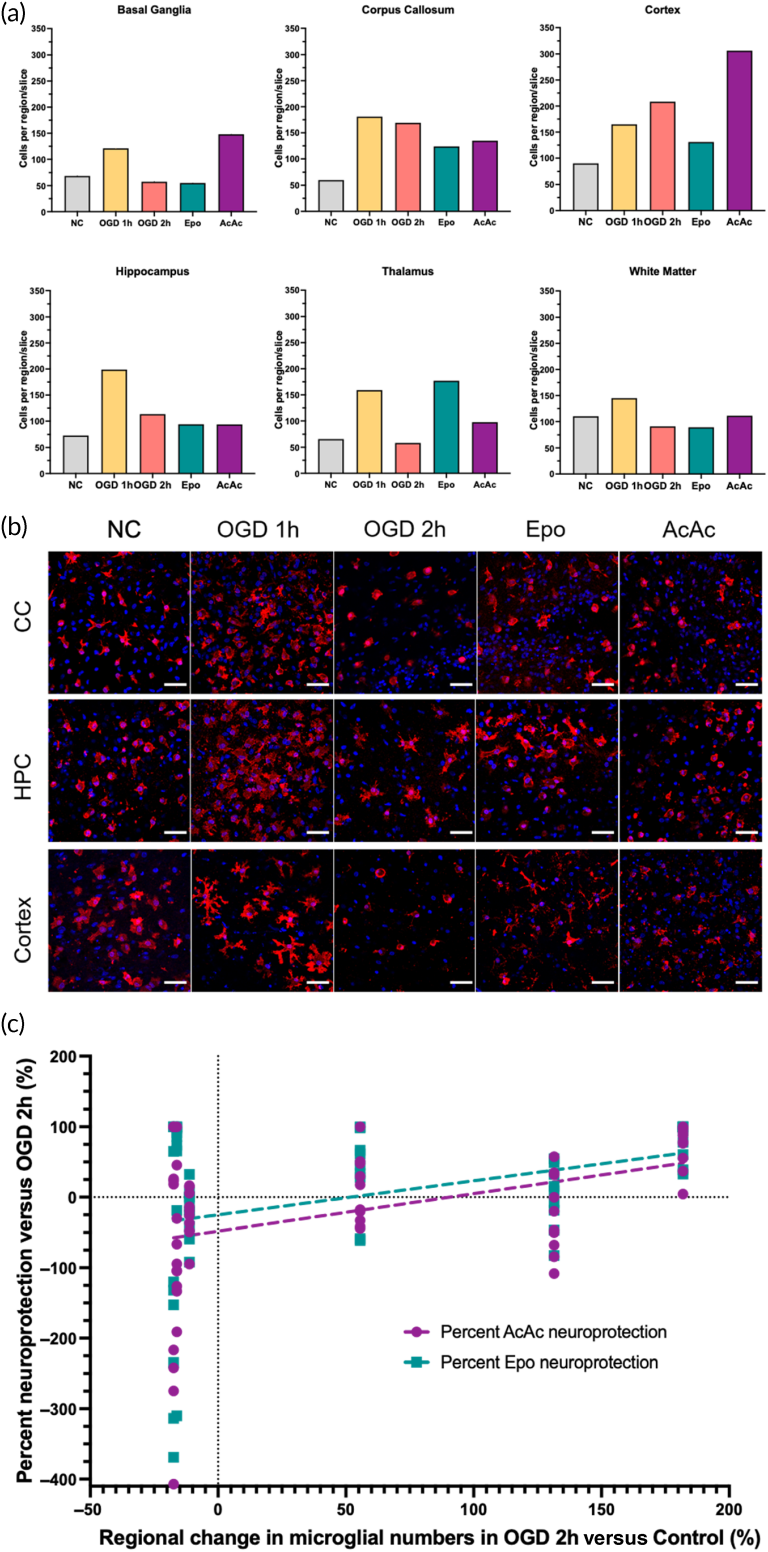
Microglial response to oxygen–glucose deprivation (OGD) and treatment. (a) Global average microglial numbers per region/treatment. Microglial counts per region were averaged from *n* = 5–7 slices per condition (NC *n* = 7; OGD1h *n* = 5; OGD2h *n* = 6; Epo *n* = 6; AcAc *n* = 5). (b) Selected representative microglial images. In general, OGD 1 h resulted in an expansion in the number of microglial cells, which appeared to often have a hyper‐ramified appearance (e.g., in the cortex). Increasing OGD to 2 h generally reduced the number of microglial cells again, but this was not consistent across regions. For instance, in the cortex microglia number increased further, which increased again with AcAc treatment. Similar heterogeneity was seen with response to Epo, such as a reduction in microglial number in the cortex, but an increase in the thalamus. Scale bars are 50 μm in all images. (c) Linear regression of percent neuroprotection in each region of each slice (relative to median percent PI‐positive cells in OGD 2 h group) versus the average change in microglial number in that region in response to OGD. Both treatments had increased neuroprotection in regions that saw a greater increase in microglial numbers in that region (Epo *R* ^2^ = 0.17, *p* = 0.001; AcAc *R* ^2^ = 0.12, *p* = 0.01)

Microglial counts per region were averaged from *n* = 5–7 slices per condition (NC *n* = 7; OGD1h *n* = 5; OGD2h *n* = 6; Epo *n* = 6; AcAc *n* = 5). Region‐specific changes in the estimated number of Iba‐1+ cells per ROI per region/slice were highly dependent on treatment group (Figure 4). In general, the OGD 1 h group appeared to have an increase in total microglia across all regions, though this effect was smallest in the subcortical white matter (Figure 4a). Increasing OGD time to 2 h tended to reduce microglial numbers to levels similar to those seen in the control group, except for in the corpus callosum and cortex. In these regions, Epo treatment after 2 h OGD then normalized cell numbers back to control levels. AcAc appeared to increase the number of microglia in the basal ganglia and cortex, with opposite effects of Epo and AcAc seen in the thalamus (increases due to Epo, decreases due to AcAc). Representative Iba‐1 images from selected regions are shown in Figure 4b. In OGD 2 h slices, compared to control slices, no associations between absolute or relative regional changes in microglial number and absolute or relative changes in injury (percent PI‐positive cells) were seen (data not shown). When examining the magnitude of region‐specific neuroprotection provided by Epo and AcAc, in general, the degree of neuroprotection by both compounds was positively correlated with the change in microglial numbers after 2 h of OGD relative to control, though the variability was high (Epo *R* ^2^ = 0.17, *p* = 0.001; AcAc *R* ^2^ = 0.12, *p* = 0.01; Figure 4c). Two notable exceptions were the basal ganglia and subcortical white matter, where microglial number decreased below control levels in the OGD 2 h group and opposing effects of the two treatments were seen (Supplemental Figure 3). In the basal ganglia, microglia numbers decreased by 16% in the OGD 2 h group compared to control slices, with Epo treatment reducing PI‐positive cells by a median of 86% and AcAc increasing PI‐positive cells by a median of 99% (Supplemental Figure 3A). Conversely, in the subcortical white matter, microglia numbers decreased by 17% in the OGD 2 h group compared to control slices, with Epo treatment increasing PI‐positive cells by a median of 132% and AcAc decreasing PI‐positive cells by a median of 21% (Supplemental Figure 3B). Response to both therapies also appear to be more variable in the white matter than in the basal ganglia.

### 3.7 Global microglial morphology responses to injury and treatment

**FIGURE 5:**
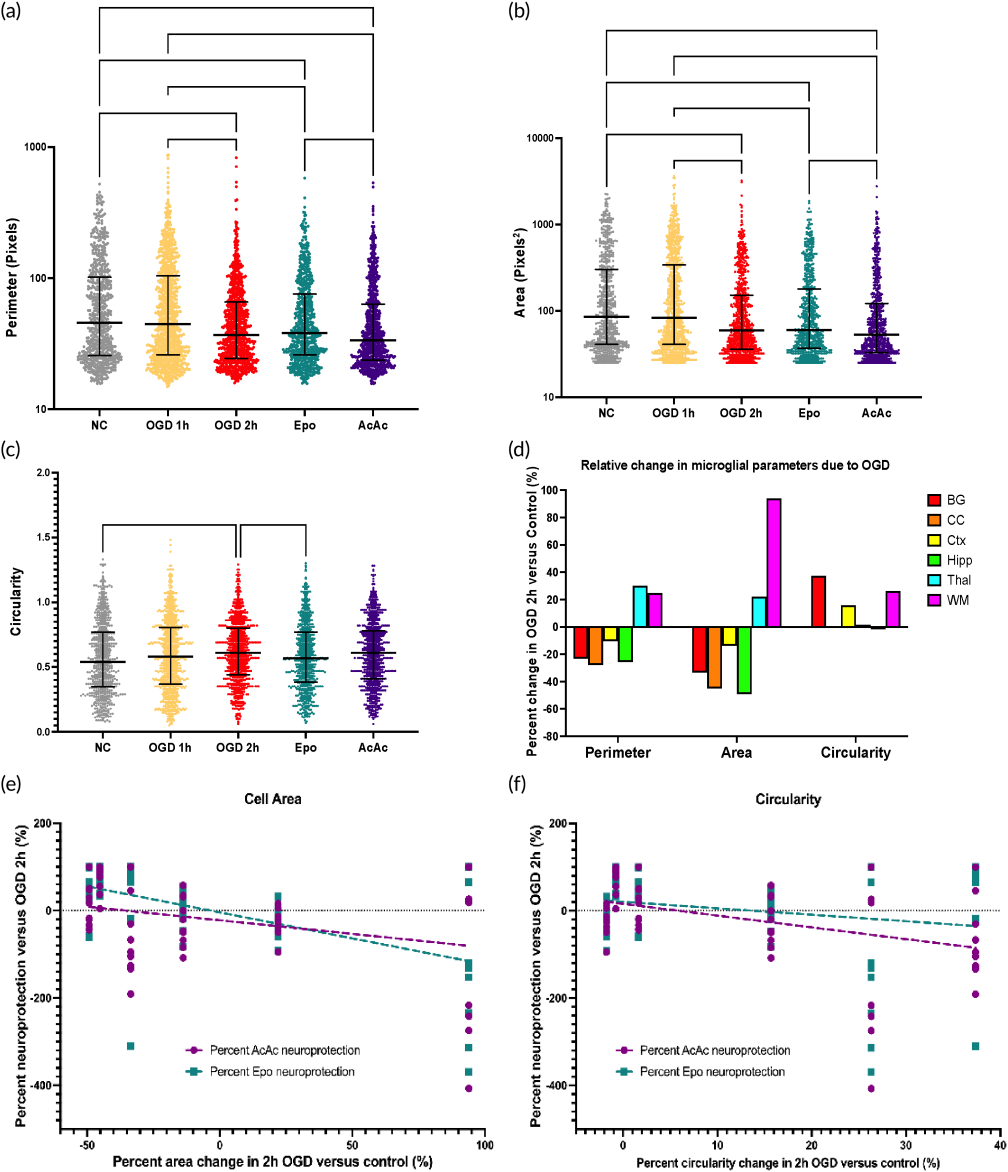
Microglial shape parameters by group and correlation between relative change in microglial morphology parameters after 2 h oxygen–glucose deprivation (OGD) and neuroprotection by Epo and AcAc. Microglial perimeter (a), area coverage (b), and circularity (c) by treatment group. Graphs display median with interquartile range. OGD 1 h did not result in any significant changes in microglial morphology. OGD 2 h resulted in an average decrease in area and perimeter, with a corresponding increase in circularity. Epo treatment significantly reduced circularity compared to OGD 2 h. Treatment with AcAc resulted in area coverage and perimeter values significantly lower than after Epo treatment. (d) Relative changes in morphology parameters in the OGD 2 h slices compared to control slices, by region. Decreases in perimeter and area coverage were seen in the basal ganglia, corpus callosum, cortex, and hippocampus, with circularity increasing in the basal ganglia and cortex. Conversely, perimeter and area coverage increased in the thalamus and subcortical white matter in OGD 2 h slices, with circularity also increasing in the white matter. Minimal changes in circularity were seen in the corpus callosum, hippocampus, and thalamus. (e) Changes in microglial area coverage in response to OGD (compared to control) were significantly correlated with degree of neuroprotection in the Epo group (*R* ^2^ = 0.29, *p* < 0.0001). (f) Changes in microglial circularity in response to OGD better‐predicted neuroprotection by AcAc (*R* ^2^ = 0.16, *p* = 0.002), with no association seen with response to Epo. *(*p* < 0.05), ** (*p* < 0.01), and *** (*p* < 0.001) indicate significant difference with Kruskal–Wallace test adjusted for multiple comparisons

Overall, 1 h OGD did not meaningfully alter global microglial morphology compared to control (Figure 5a–c). Therefore, morphology in the 1 h OGD group was not considered in further analyses. By comparison, 2 h OGD significantly decreased both cell perimeter (Figure 5a) and cell area coverage (Figure 5b), and increased cell circularity (Figure 5c), all of which individually may be associated with a phenotypic switch to more activated microglia.^17^, ^18^, ^19^ Treatment with AcAc did not change any morphological parameters compared to 2 h OGD, but treatment with Epo did significantly reduce circularity (Figure 5a–c).

### 3.8 Regional microglial morphology responses to injury and treatment

Significant regional variability in microglial morphology was seen in both the control slices and response to 2 h OGD (Figure 4B, Supplemental Figure 4A). We examined regional morphology in control and OGD slices separately because of these differences. In control microglia, cell perimeter tended to be shortest in the cortex and thalamus, but no significant differences in perimeter were seen across regions (Supplemental Figure 4A). After 2 h OGD, microglia in the basal ganglia had significantly longer perimeters compared to cortical microglia, and white matter microglia had significantly longer perimeters than microglia in the corpus callosum, cortex, and hippocampus (Supplemental Figure 4B). Similar to cell perimeter, cell area coverage was also lowest in the cortex and thalamus in control slices, with cortical microglia cell area coverage significantly smaller than cell area coverage in the basal ganglia and hippocampus (Supplemental Figure 4C). Changes in area coverage after 2 h OGD mirrored changes seen in perimeter (Supplemental Figure 4D). In control slices, greater regional variability in circularity was seen compared to the other parameters; cell circularity was greatest in control corpus callosum and hippocampus, with microglia in the corpus callosum significantly more circular than the basal ganglia, cortex, and white matter, and hippocampal microglia also significantly more circular than microglia in the white matter (Supplemental Figure 4E). After 2 h OGD, these regional differences in circularity were largely eliminated, with no significant differences seen across regions (Supplemental Figure 4F).

When examined as regional changes relative to control slices, in the basal ganglia, corpus callosum, cortex, and hippocampus, OGD 2 h resulted in relative decreases in perimeter and area coverage, with circularity increasing in the basal ganglia and cortex (Figure 5d). Conversely, perimeter and area coverage increased in the thalamus and subcortical white matter in OGD 2 h slices, with circularity also increasing in the white matter (Figure 5d). Minimal changes in circularity were seen in the corpus callosum, hippocampus, and thalamus. Regional changes in microglia area coverage in response to OGD (compared to control) were significantly correlated with degree of neuroprotection seen with treatment—with decreases in cell area coverage associated with neuroprotection—though the relationship was more meaningful in the Epo group (*R* ^2^ = 0.29, *p* < 0.0001) than the AcAc group (*R* ^2^ = 0.1, *p* = 0.16; Figure 5e). By comparison, regional changes in microglial circularity in response to OGD better predicted neuroprotection by AcAc (*R* ^2^ = 0.16, *p* = 0.002), with neuroprotection seen in regions where circularity did not change in response to OGD, and with no association seen with response to Epo (*R* ^2^ = 0.04, *p* = 0.15; Figure 5f).

### 3.9 Microglial SM responses to injury and treatment

**FIGURE 6:**
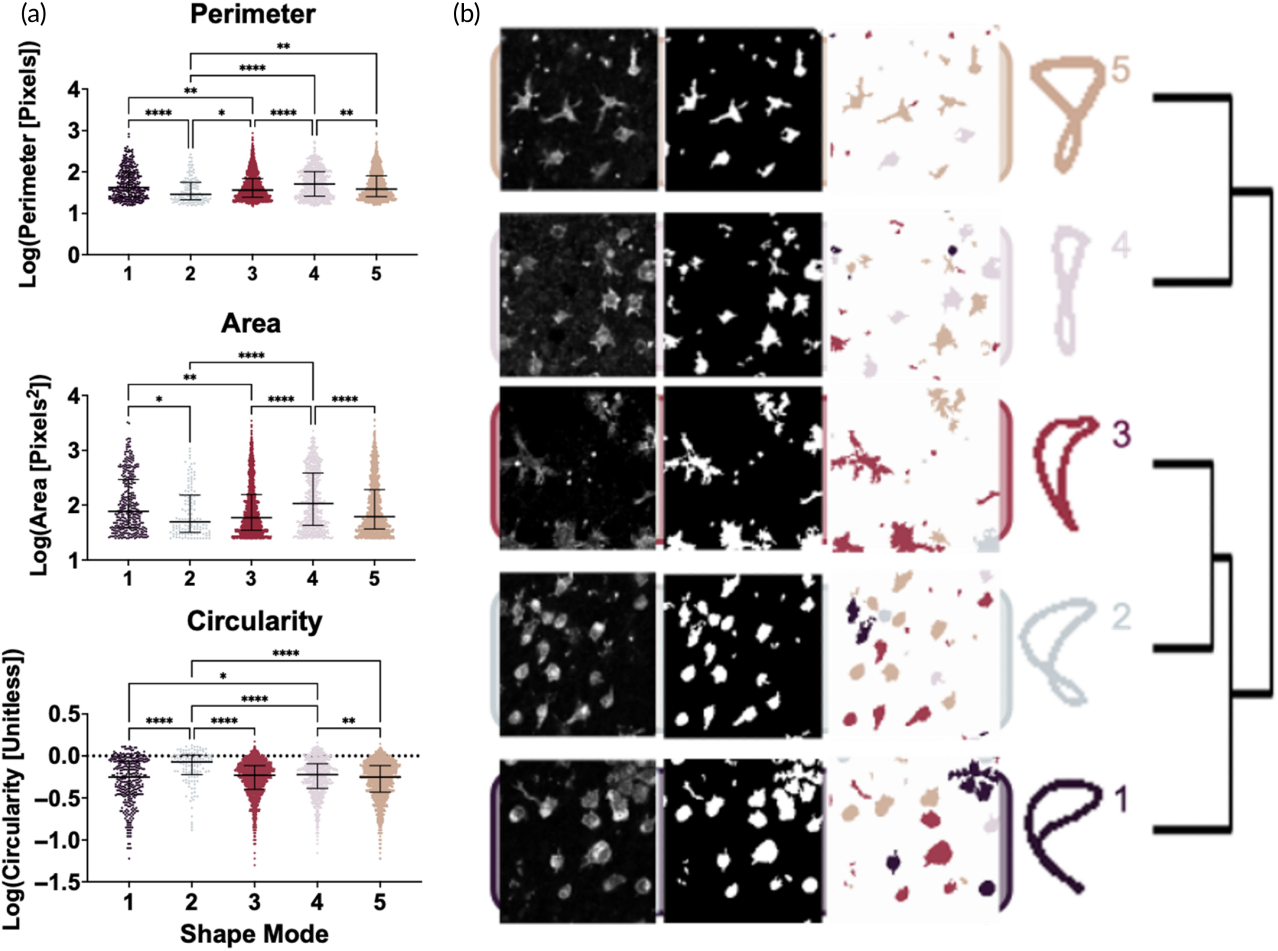
Cell morphology parameters by shape mode and odds of shape mode by sex, region, and region by treatment interactions. (a) Morphology parameters for the five VAMPIRE‐derived SMs. Graphs depict median with interquartile range. * (*p* < 0.05), ** (*p* < 0.01), and **** (*p* < 0.0001) indicate significant difference with Kruskal–Wallace test adjusted for multiple comparisons. (b) Segmentation and labeling procedure with representative SM images (colored according to SM on right), including shape mode projections after k‐means clustering

The five VAMPIRE‐derived SMs differed from one another significantly in most of the morphological parameters (Figure 6a). Perimeter was longest in SM4 and shortest in SM2, which also corresponded to the largest and smallest area coverage, respectively. Circularity was also highest in SM2 but similar across the other SMs. The segmentation procedure and representative SM images are shown in Figure 6b. Proportions of each SM in each region of control and OGD slices are shown in supplemental Figure 5A‐B. In control slices, the proportion of SM2 microglia was positively correlated with number of PI‐positive cells, and proportions of SM3 and SM4 negatively correlated with percentage of PI‐positive cells (Supplemental Figure 5C). In OGD slices, the proportion of SM4 and SM5 microglia were both positively correlated with number of PI‐positive cells (Supplemental Figure 5D).

**FIGURE 7:**
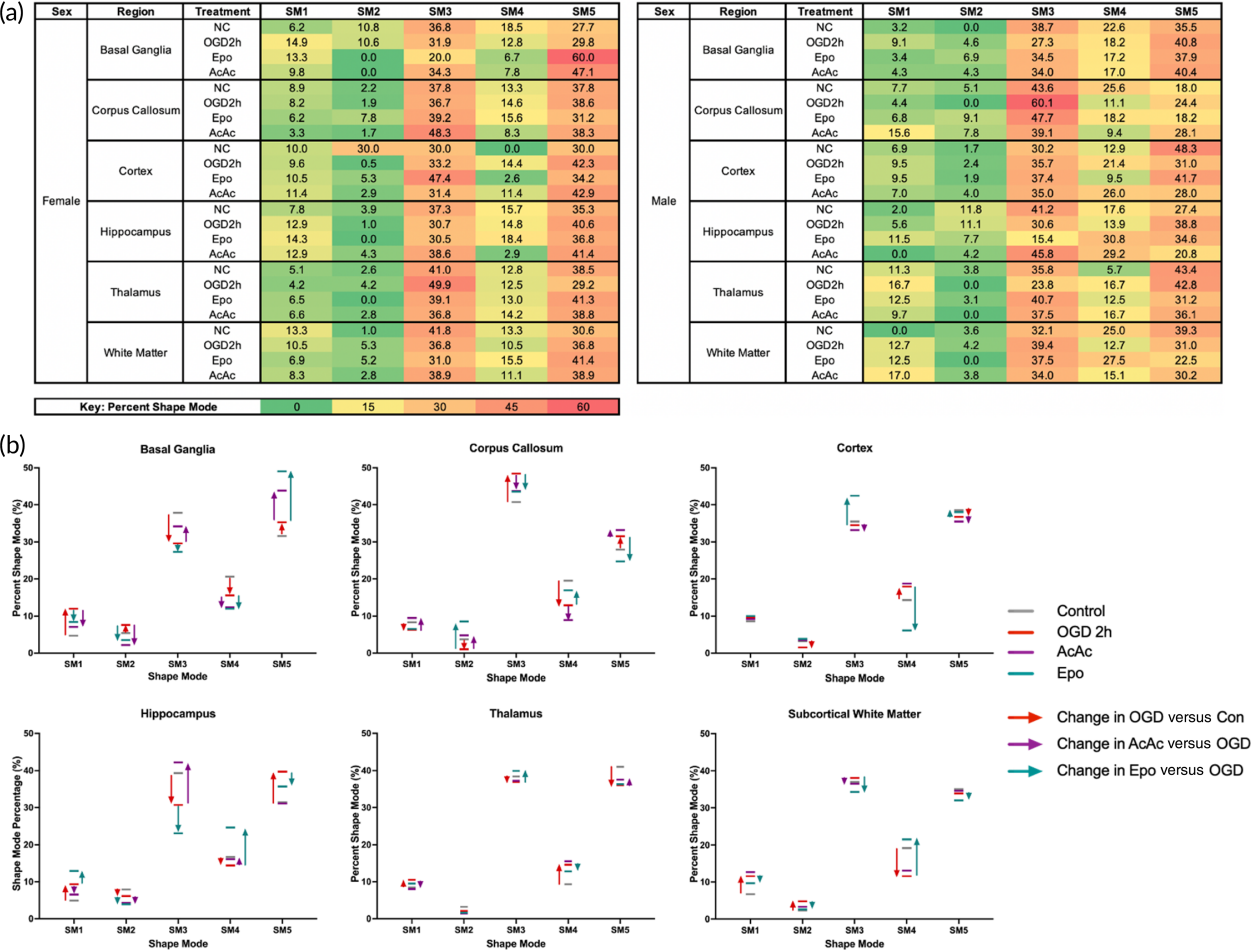
Shape mode changes after oxygen–glucose deprivation (OGD) and treatment. (a) Global heat maps of percent SM by sex, region, and treatment group. (b) Changes in relative proportion of each SM as a result of OGD (compared to control) and treatment with Epo or AcAc (compared to OGD 2 h). Across all regions, SMs 3 and 5 were the most common regardless of treatment. SMs 3–5 generally saw the largest differences across treatment groups in all regions, though minimal overall change in any SM was seen in the thalamus

A global heatmap for percent SM by sex, region, and treatment group is shown in Figure 7a. Global sex‐agnostic alterations in relative proportion of each SM as a result of OGD (compared to control) and treatment with Epo or AcAc (compared to OGD 2 h) are depicted in Figure 7c,d. Across all regions, SMs 3 and 5 were the most common regardless of treatment. SMs 3–5 generally saw the largest differences across treatment groups in all regions, though minimal overall change in any SM was seen in the thalamus. Logistic regression contrasts by sex of the slice showed that male microglia had significantly greater odds of being SM4, and decreased odds of being SM5, compared to female slices (Supplemental Table 1). Regional differences in SM were also most pronounced with SMs 4 and 5; with the cortex as the reference region, microglia in the basal ganglia showed significantly increased odds of being SM4, and decreased odds of being SM5. Microglia in the corpus callosum, hippocampus, and white matter also had a significantly lower odds of being SM5 compared to microglia in the cortex. Epo and AcAc often had contrasting effects on odds of a given SM or had opposite effects on a given SM within different regions. For instance, when compared to OGD 2 h, AcAc resulted in decreased odds of SM2 in the basal ganglia, while Epo had increased odds of SM2 in the corpus callosum. Epo also resulted in decreased odds of SM4 in the cortex but increased odds of SM4 in the subcortical white matter.

### 3.10 Regional microglial morphology responses to injury predict treatment response

When examining regional changes in the proportion of each SM, an increase in the proportion of SM5 after OGD compared to control appeared to correlate with increasing injury in that region (Supplemental Figure 6A). Similarly, an increase in SM5 within a given region was also associated with positive neuroprotection by Epo in that same region (Supplemental Figure 6B). In logistic regression models predicting whether a given region in a slice saw neuroprotection (e.g., had a lower percent of PI‐positive cells compared to 2 h OGD regional median), each percentage increase in proportion of SM5 cells in that region after OGD was associated with a significantly increased odds of Epo neuroprotection (OR 1.23 95% CI [1.06–1.43]). This proportion change in SM5 in that region predicted regional neuroprotection by Epo with an AUROC of 0.71 (Table 2). No other SM changes were associated with Epo neuroprotection (data not shown). For AcAc, the effect of each individual SM was smaller but overall, still predictive; increases in SM1 and SM4 were negatively associated with neuroprotection and increases in SM5 associated with increases in neuroprotection (Table 2). When combining changes in SM1, SM4, and SM5 into a model, regional neuroprotection by AcAc was predicted with an AUROC of 0.81.

**TABLE 2:** Predicting regional Epo and AcAc neuroprotection by shape mode responses to OGD

Epo	OR of neuroprotection in region (95% CI)	AUROC	*p* value
SM5	1.23 (1.06–1.43)	0.71	0.007

AcAc	OR of neuroprotection in region (95% CI)		*p*‐value
SM1	0.80 (0.66–0.97)	0.65	0.020
SM4	0.91 (0.84–0.99)	0.67	0.024
SM5	1.13 (1.01–1.28)	0.62	0.041
SM1 + SM4		0.79	<0.0001
SM1 + SM4 + SM5		0.81	<0.0001

*Note*: Logistic regression models examining regional changes in microglial shape mode (SM) after OGD 2 h to predict whether a given region in each slice saw neuroprotection (percent of PI‐positive cells less than the median in OGD 2 h slices in that region). Only SM5 changes were associated with Epo neuroprotection, with SM5 shifts alone predicting Epo neuroprotection with an AUROC of 0.71. Smaller individual effects of SM2 were seen for AcAc neuroprotection, but a combined model including changes in SM1, SM4, and SM5 resulted in prediction of regional AcAc neuroprotection with an AUROC of 0.81.

## 4 DISCUSSION

Organotypic slice culture models, particularly using OWH slices, allow for in‐depth investigation of regional changes in response to injury and therapy, which can support mechanistic investigation of therapeutic strategies as well as high‐throughput screening of treatments before translation into larger animal models. Here, we describe the development of a term‐equivalent ferret OWH model of OGD and treatment, with particular focus on regional changes in microglial morphology and their associations with injury severity and response to treatment in order to better examine injury responses seen in vivo. We show that, even though both Epo and AcAc suppress global cell death in response to a 2 h OGD exposure, this effect is highly heterogeneous across different regions of the brain. The magnitude of regional changes in microglial staining in response to injury, as well as treatment with Epo, in vitro also mirrored more global changes seen after injury in vivo. Importantly, regional changes in both microglial morphology and VAMPIRE‐generated morphological SMs were associated with degree of injury due to OGD and therapy‐specific treatment responses, suggesting an ability to predict region‐specific treatment responses based on local cellular changes after injury.

While historical rodent brain slice culture techniques focus on a single region,^20^ we have previously shown that OWH slice culture models retain much of the three‐dimensional architecture of the intact brain while allowing for multiple analytical techniques such as live‐cell imaging in deep brain structures that are not accessible with in vivo imaging.^8^, ^9^, ^10^, ^21^ Prior work in OWH slice culture has focused on small lissencephalic brains such as those from rodents and rabbits, with larger gyrencephalic brains from human^22^, ^23^, ^24^ or nonhuman primate samples necessitating culture of slices from individual brain regions. As a small animal with a gyrencephalic brain, the ferret allows for OWH slice culture of multiple critical regions of the gyrified brain at the same time, with particular interest in the ferret coming from those examining effects on early life injury and the developing neocortex.^3^, ^4^, ^5^, ^25^ In the model presented here, term‐equivalent OWH ferret slices were exposed to 2 h OGD in the final injury model, with the corpus callosum, hippocampus, and thalamus particularly showing increases in injury (as measured by relative change in PI‐positive cells) in response to OGD, as well as nonsignificant increases in cell death in the basal ganglia. As these areas are particularly associated with common injury patterns in term infants with asphyxial brain injury resulting in HIE,^26^ this model can provide a useful ex vivo platform with which to screen therapeutics for infants with HIE.

After 2 h OGD, global injury responses (LDH release into culture media) were suppressed by treatment with either Epo or AcAc. Although AcAc appeared to result in greater suppression of LDH release, regional PI staining appeared to be more significantly improved by Epo, with the discrepancy perhaps being due to the relative timing and type of cell death accounted for by the two assessments. Both treatments also increased total GSH levels after OGD, but only Epo recovered reduced GSH levels. This suggests that, while AcAc is able to mitigate an injury‐induced reduction in intracellular GSH, it does not prevent the oxidative stress associated with injury, and as a result, the cellular GSH remains relatively oxidized compared to Epo, which appears to have more direct anti‐oxidative properties. Similarly, Epo appeared to normalize mitochondrial morphology in response to OGD—either due to prevention of fission or stimulation of fusion—with more modest improvements after treatment with AcAc. Both Epo and AcAc also normalized expression of a number of genes responsive to both inflammation and oxidative stress, though the AcAc slices appeared to have global suppression of all assessed transcripts below control, particularly ones associated with oxidative stress such as CHAC1 and SLC7A11. This dose of AcAc has previously been shown to be neuroprotective and mitoprotective in vitro,^12^ particularly by suppressing activation of the mitochondrial permeability transition pore. Whether this is part of the neuroprotective effect of AcAc, or specific to the ferret and OWH model used here, remains to be seen. Importantly, while there was one outlier for the majority of the transcripts in the 2 h OGD group, our use of nonparametric statistics means that the absolute value from this outlier will not have affected the overall results. Overall, the ferret OWH platform allows for the investigation of multiple mechanistic downstream effects of therapy in addition to region‐specific imaging. The fact that AcAc and Epo resulted in differing regional and global effects supports the idea that combinatorial therapies, rather than single strategies, could provide optimal global neuroprotection,^27^ with the ferret OWH slice culture model providing a platform with which to assess multiple therapeutic combinations.

Similar to the differences in injury response, microglial changes in response to OGD were also highly heterogeneous across brain regions. Importantly, though Iba‐1 is a nonspecific marker of myeloid cells including macrophages in addition to microglia,^28^ it is likely that the vast majority of stained cells in the OWH slice culture model are microglia, with any macrophages present being only those that were in the vasculature when the brain was harvested. However, this would not necessarily be the case for the in vivo model data, where Iba‐1 staining could include penetrating peripheral macrophages. In general, 1 h of OGD resulted in an expansion in the number of microglia across all brain regions, with 2 h of OGD then tending to decrease numbers back to control levels, except in the corpus callosum (no further change) and cortex (slight increase). After treatment, Epo tended to normalize any increases in microglial number, except in the thalamus where an increase was seen. AcAc resulted in increases in microglial numbers in the basal ganglia and cortex, with normalization or no effect elsewhere.

The regional responses to OGD and Epo largely mirror our own preliminary evidence examining regional Iba‐1 staining intensity after in vivo injury. For instance, control Iba‐1 staining in the cortex was around twice that of the corpus callosum, mirroring the differences in total microglial number in those same regions of control OWH slices. Relative increases were then much larger in the corpus callosum both in vivo and in vitro after injury, with Epo only being meaningfully associated with improvement (and neuroprotection) in the corpus callosum. This suggests that regional myeloid cell staining intensity several weeks after injury in vivo shows similar responses to injury and treatment as that seen in vitro, again supporting the slice culture model as a platform for therapeutic screening and mechanistic investigation. However, a more direct comparison of regional responses to treatment both in vitro and in vivo, including whether regional responses to therapy correlate with improvements in behavior, will be required. As an example, we have recently published long‐term outcomes after Epo treatment in the in vivo model, including behavioral assessments.^29^ Interestingly, Epo treatment resulted in significant improvements in multiple neuropathological assessments but was less clearly associated with improvements in motor function. Future work will include more direct comparison of regional effects of multiple treatments both in vitro and in vivo, including more complex behavioral tasks such as social interaction and operant conditioning.^30^, ^31^

Although the relative change and absolute number of microglia were not associated with the injury response to 2 h of OGD, in general both Epo and AcAc were more neuroprotective in regions that saw expansions in the number of microglia in response to OGD. Recently, McDonough et al. showed that ischemic preconditioning, which is neuroprotective against multiple forms of acute brain injury, results in the expansion of cortical microglia.^32^ Similarly after spinal cord injury, expanded microglial populations facilitate repair via scar generation.^33^ Both studies suggest that an expanded microglial population may be required for later neuroprotective responses in models that consist of HI‐reperfusion injury. However, further mechanistic investigation of this effect is required in future studies, including investigation of why the basal ganglia and subcortical white matter were outliers for both treatments: we saw relative neuroprotection by Epo and increased injury by AcAc in the basal ganglia, and relative neuroprotection by AcAc and increased injury by Epo in the white matter.

With the recent focus on microglia as critical components of response to acute brain injury and risk for chronic neurodegeneration, assessment of microglial phenotype has encompassed a range of increasingly‐targeted techniques.^34^ Regional heterogeneity in microglial morphology and transcriptional phenotype has already been extensively described in both the rodent and human brain,^35^ with both canonical and noncanonical microglial subtypes described.^36^ These microglia have different specific morphology and functions, and also result in increased complexity of subtypes as a result of injury or in the setting of neurodegeneration, where response may include proliferation, phenotypic shifts, or minimal response.^36^ The regional heterogeneity in microglial structure and response to OGD in our study is, therefore, not unexpected. Global changes in morphology in response to OGD resulted in classical “activated” or ameboid microglial morphology, with a decrease in area of coverage and cell perimeter, and increase in cell circularity.^17^, ^18^, ^19^ Opposite effects were seen in areas such as the thalamus and white matter, suggesting a “hyper‐ramified” morphology taking precedence in the setting of injury in those regions. These morphological changes were also correlated with treatment‐specific heterogeneity in regional neuroprotection; Epo was more neuroprotective in areas where microglia showed a classical activation response by decreasing cell area coverage, whereas AcAc was only neuroprotective when minimal morphological change in microglia was seen. These differences in response to treatment based on microglial changes are also not unexpected. For instance, Epo is thought to have pleiotropic effects including being anti‐inflammatory,^37^, ^38^, ^39^ which may explain the greater relative neuroprotection in brain regions displaying more classical activation of microglia. Therefore, Epo may be beneficial only once the injury or degree of inflammation after injury is sufficiently severe, an effect potentially similar to TH, the only established therapy for neonatal HIE. TH has so far only been shown to be neuroprotective in moderate‐to‐severe injury^40^; however, this is largely due to the lack of adequate clinical trials in infants with mild HIE. By comparison, having at least some degree of microglial inflammatory response is necessary for recovery from injury in multiple models of neurological injury including in the developing brain,^41^, ^42^, ^43^ with AcAc perhaps being neuroprotective when a microglial inflammatory response does not happen. The hope is that, once better‐established in the preclinical setting, clinical correlates of scenarios that dictate these differential regional responses to injury and treatment can together improve development of therapies. These data, therefore, show that further research into the regional heterogeneity of microglia subtypes is essential to better understand injury trajectories, which could improve the targeting of therapeutics based on the location of the injury. It is also important to note that, while relatively modest *R* ^2^ values for linear regressions between changes in microglial morphology after OGD and later response to therapy were seen, these can still be considered both useful and meaningful. For instance, regional changes in area of cell coverage after OGD predicted 29% of the regional variability of response to Epo, and changes in circularity predicted 16% of the variability in response to AcAc. While a large proportion of variability remains unaccounted for, we find it promising that these simple parameter changes may help to predict a significant proportion of the regional variability in response to treatment.

In an attempt to categorize microglia morphology in OWH ferret slice culture, we adapted the VAMPIRE methodology, which was initially developed to classify morphological heterogeneity of cancerous cells,^15^ for use with microglial images. The VAMPIRE platform is an unsupervised machine‐learning method that extracts contour coordinates, normalizes and aligns shapes, reconstructs object shapes after principal component analysis of the coordinates, and then identifies a prespecified number of SMs based on k‐means clustering.^15^ Generating five separate SMs allowed for identification of morphological groupings of cells across regions that at least partially explained regional responses to injury and treatment. This was particularly the case for SM5, which tended to increase in regions that saw more injury in response to OGD, as well as predicting the majority of the neuroprotective effect of Epo. Although SM 5 displayed smaller area of coverage and cell perimeter than other SMs (particularly SMs 1 and 4), it also had relatively low median circularity and was one of the most common SMs—contributing 30%–50% of Iba‐1 positive cells depending on region/condition. Although additional future work is required, the VAMPIRE method of morphological grouping identifies morphologic features beyond the typical ways in which microglia have been classified. For instance, it is clear that a high degree of variability in individual morphological parameters is seen within each of the SMs. The goal of the VAMPIRE analysis was not to group cells based on those individual parameters but to examine the importance of morphological characteristics beyond the more classical shapes described in the literature. As both canonical morphological phenotypic changes (e.g., circularity) and SM shifts both predicted degree of injury and response to therapy in different ways, this suggests that methods such as VAMPIRE can allow us to identify microglial subtypes that could be described based on features that are not necessarily identifiable using other methods while being complementary in terms of finding ways to classify microglia and predict responses to therapy. Future studies will include regional microdissection after culture, OGD, and treatment, in order to align SM subtypes with transcriptional and biochemical phenotyping, as has previously been performed with VAMPIRE analyses in tumor cells.^15^ Recently, microglia from mouse slice culture has been isolated and shown to be transcriptionally similar to those seen in vivo,^44^ providing evidence to support the idea that OWH slice culture can help us understand the intact brain environment.

This study has several limitations. Although slice culture conditions were tightly controlled across outcome measures, comparisons of regional PI staining intensity and microglial changes were based on data from separate slices. Global numbers of microglia per region in each treatment group could also only be provided as a single average (total cells from test set/number of slices). Due to the nature of the VAMPIRE code in addition to fully blinding the images for analysis, we were unable to trace each cell count from the randomized test dataset back to the specific slice it came from; however, this code has been rewritten to allow for this to be done in the future. In addition, while sex‐specific differences in both microglial phenotype and response to injury and treatment are expected in the developing brain,^35^, ^43^, ^45^ we did not have a large enough sample size or resulting statistical power to formally model sex * group * region interactions. However, the fact that region/group‐agnostic analyses suggested differences in SM 4/5 prevalence in male/female brains, as well as the fact that different cell death pathways are recruited based on sex, indicate that this platform may be helpful for eliciting sex‐specific injury mechanisms and therapies in the future. Importantly, regional heterogeneity in microglial and injury responses to OGD and treatment cannot exclusively be attributed to inherent regional differences in vivo. As PI staining intensity was also variable across regions, it may be that some of the changes seen were due to different tolerances in regions to the process of slicing and culturing. Comparison of microglial phenotypes in vivo and in vitro in the future may help to discern what can be attributed to the process of culturing itself. Finally, the selection of five SMs, as was done during initial VAMPIRE development, is unlikely to capture all the microglial heterogeneity seen in the brain, as more subtypes of microglia than this have been described in the literature.^36^, ^46^

## 5 CONCLUSION

In summary, we show that OWH slice culture of the term‐equivalent brain is feasible in the ferret and allows for multiple biochemical and morphological analyses of regional heterogeneity in response to injury and treatment. Our results support the OWH brain slice model as an ideal platform with which to collect high‐dimensionality biological data amenable to advanced data science techniques while remaining relevant to the in vivo environment. Machine‐learning‐based classification of microglial phenotypic shifts in response to injury predicted the majority of the neuroprotective response to therapy, with different subtypes associated with different treatment responses. Regional differences in global microglial response to injury and treatment also mirrored those seen after injury in vivo. This suggests that the ferret OWH slice culture model can provide a powerful tool in the process of examining regional responses to injury in the gyrencephalic brain, as well as for screening combinations of therapeutics to provide global neuroprotection after injury.

## CONFLICT OF INTEREST

The authors declare that they have no relevant conflicts of interest.

## AUTHOR CONTRIBUTIONS

**Thomas R. Wood:** Conceptualization (equal); data curation (equal); formal analysis (lead); funding acquisition (equal); investigation (equal); resources (equal); supervision (equal); validation (lead); visualization (equal); writing – original draft (equal); writing – review and editing (lead). **Kate Hildahl:** Data curation (equal); investigation (equal); methodology (lead); validation (equal); visualization (equal); writing – original draft (equal). **Hawley Helmbrecht:** Data curation (equal); investigation (equal); methodology (equal); software (lead); visualization (equal). **Kylie A. Corry:** Data curation (equal); investigation (supporting). **Daniel H. Moralejo:** Data curation (supporting); investigation (supporting); methodology (equal). **Sarah E. Kolnik:** Data curation (supporting); investigation (supporting); methodology (supporting). **Katherine E. Prater:** Formal analysis (supporting); validation (equal); writing – review and editing (supporting). **Sandra E. Juul:** Funding acquisition (supporting); resources (supporting); validation (supporting); writing – review and editing (supporting). **Elizabeth Nance:** Conceptualization (lead); formal analysis (equal); funding acquisition (equal); project administration (lead); resources (equal); supervision (equal); validation (equal); visualization (supporting); writing – review and editing (equal).

### PEER REVIEW

The peer review history for this article is available at https://publons.com/publon/10.1002/btm2.10265.

## Supporting information


**Appendix** **S1**. Supporting Information.

## Data Availability

The data that support the findings of this study are available from the corresponding author upon reasonable request.
