# Supplementary material for: A ferret brain slice model of oxygen–glucose deprivation captures regional responses to perinatal injury and treatment associated with specific microglial phenotypes
Source: Bioeng Transl Med. 2021 Nov 23;7(2):e10265. doi: 10.1002/btm2.10265 (PMC9115703; doi:10.1002/btm2.10265)
Supplement: Supplementary file 1 — Appendix S1. Supporting Information. [file BTM2-7-e10265-s001.docx]

**Supplemental Material**


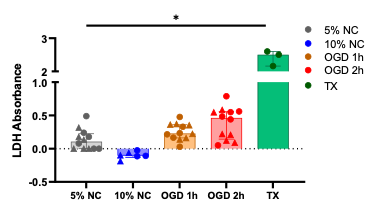

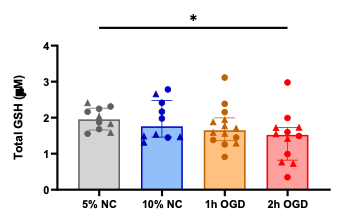

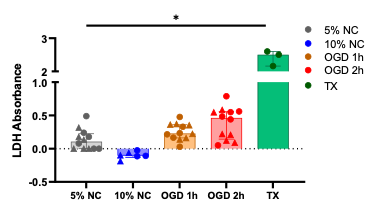


A

B

**Supplemental Figure 1.** LDH release **(A)** and total GSH **(B)** in slices during optimization of culturing conditions. LDH release was compared to TritonX (TX) lysed control. Using 10% serum resulted in negative LDH measurements due to interference with the assay. OGD 1h did not significantly increase LDH release or result in depletion of GSH, both of which were significantly altered by OGD 2h. *Indicates significant difference (p<0.05) with Kruskal-Wallace test after adjustment for multiple comparisons.


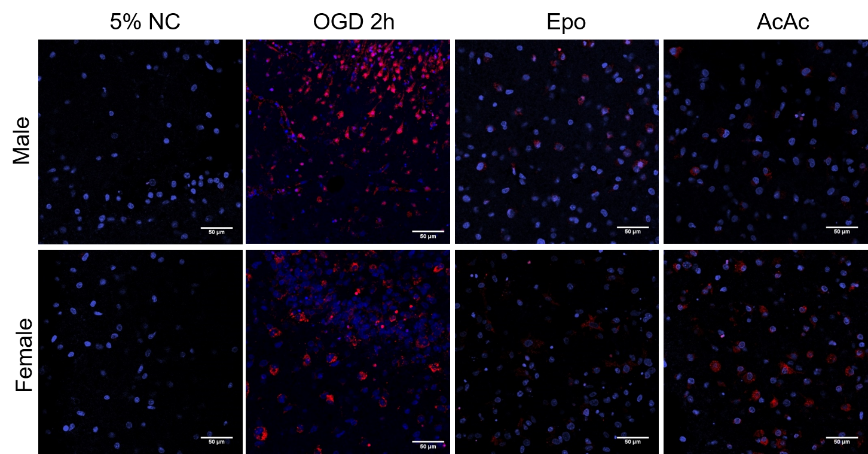


**Supplemental Figure 2. Representative PI staining by treatment group and sex.** Representative PI staining of hippocampus in the injury and treament groups by sex. PI positive cells were counted as those where clear red staining was seen in the nucleus.

**Supplemental Figure 3. Divergent regional treatment responses despite comparable changes in microglial number.** In both the basal ganglia **(A)** and subcortical white matter **(B)**, changes in total microglial number after 2h OGD was similar, with 16% and 17% reductions, respectively. Despite this, divergent responses to the therapies were seen, with net neuroprotection by Epo in the basal ganglia and a potential increase in injury in the white matter (negative neuroprotection based on PI positive cells). Conversely, AcAc increased injury in the basal ganglia, with minimal overall effect in the white matter. Responses to treatment also appeared to be more variable in the white matter, regardless of exposure to Epo or AcAc.

**Supplemental Figure 4. Regional parameter differences in control and OGD slices.** Regional changes in microglial perimeter, area coverage, and circularity in control **(A,C,E)** and OGD 2h **(B,D,F)** slices. In control microglia, cell perimeter tended to be shortest in the cortex and thalamus, but no significant differences in perimeter were seen across regions **(A)**. After 2h OGD, microglia in the basal ganglia had significantly longer perimeters compared to cortical microglia, and white matter microglia had significantly longer perimeters than microglia in the corpus callosum, cortex, and hippocampus **(B)**. Cell area coverage was also lowest in the cortex and thalamus in control slices, with cortical microglial cell area coverage significantly smaller than cell area coverage in the basal ganglia and hippocampus **(C)**. Changes in area coverage after 2h OGD mirrored changes seen in perimeter **(D)**. In control slices, greater regional variability in circularity was seen compared to the other parameters; cell circularity was greatest in in control corpus callosum and hippocampus, with microglia in the corpus callosum significantly more circular than the basal ganglia, cortex, and white matter, and hippocampal microglial also significantly more circular than microglia in the white matter **(E)**. After 2h OGD, these regional differences in circularity were largely canceled-out, with no significant differences seen across regions **(F)**. * (p<0.05), ** (p<0.01), and *** (p<0.001) indicate significant difference with Kruskal-Wallace test adjusted for multiple comparisons.

**Supplemental Figure 5. Shape mode frequencies by region and PI positive cells by shape mode frequency.** Proportions of each SM in each region of control **(A)** and OGD **(B)** slices. Notable changes in response to OGD include a loss of SM2 in the cortex and increase in SM3 in the corpus callosum. In control slices **(C)**, the proportion of SM2 microglia was positively correlated with number of PI positive cells (R^2^=0.16, p=0.006), and proportions of SM3 (R^2^=0.15, p=0.007) and SM4 (R^2^=0.15, p=0.007) negatively correlated with percentage of PI-positive cells. In OGD 2h slices **(D)**, the proportions of SM4 (R^2^=0.09, p=0.03) and SM5 (R^2^=0.12, p=0.02) microglia were positively correlated with number of PI positive cells.

**
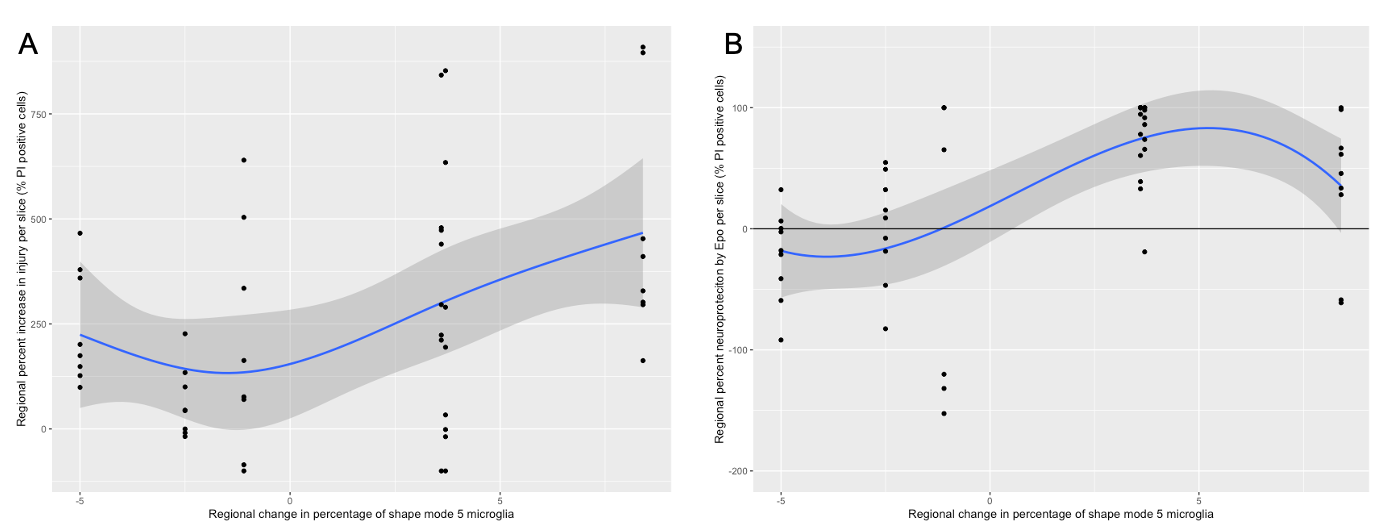
**

**Supplemental Figure 6. Shape mode 5 changes correlate with injury from OGD and response to Epo.** **(A)** Regional changes in proportion of SM5 after OGD compared to percent injury in that region. In regions where the proportion change in SM5 was positive, greater increases appeared to correlate with increasing injury. **(B)** Regional changes in proportion of SM5 after OGD compared to percent neuroprotection by Epo in that region. An increase in SM5 within a given region was associated with positive neuroprotection by Epo in that same region.

**Supplemental Table 1. Logistic Regression contrasts of shape mode by treatment group and region.** Logistic regression contrasts agnostic to other variables to determine shape mode odds by sex, treatment group (with control slices as the reference) and region (with the cortex as the reference). Significant odds ratios (with 95% CI) are bolded.

**
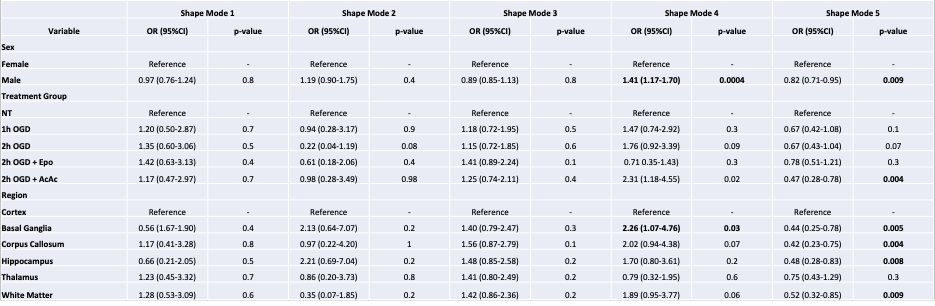
**
